# Musculoskeletal disorders and work ability of workers at a university campus in southern Brazil

**DOI:** 10.47626/1679-4435-2021-901

**Published:** 2024-02-16

**Authors:** Juliano Figueira da Silva, Adriane Vieira, Bruna Nichele da Rosa, Cláudia Tarragô Candotti

**Affiliations:** 1 Escola de Educação Física, Fisioterapia e Dança, Universidade Federal do Rio Grande do Sul, Porto Alegre, RS, Brazil

**Keywords:** occupational health, musculoskeletal pain, social determinants of health, occupational risks, saúde do trabalhador, dor musculoesquelética, determinantes sociais da saúde, riscos ocupacionais

## Abstract

**Background:**

When performed in unfavorable conditions, work can lead to the development of musculoskeletal disorders and decrease in work ability.

**Objectives:**

To identify the differences between three groups of workers (professors, technicians and outsourced workers) as for the sociodemographic profile, work ability, prevalence of musculoskeletal disorders, activity restrictions, and the correlation between the presence of musculoskeletal disorders and activity restrictions with each domain of the Work Ability Index.

**Methods:**

The sample consisted of 67 university workers assessed by a Sociodemographic Data Questionnaire, the Nordic Musculoskeletal Symptoms Questionnaire and the Work Ability Index. Descriptive statistics and Kendall's Tau correlation coefficient were used.

**Results:**

Professors presented more favorable sociodemographic and lifestyle aspects and higher work ability, while outsourced workers had less favorable sociodemographic and lifestyle aspects and lower work ability. The correlation between activity restrictions and work ability was found in only one domain of Work Ability Index among professors. Among outsourced workers were found correlations on presence of musculoskeletal disorders and activity restrictions with six domains of Work Ability Index. Technicians did not show significant correlation.

**Conclusions:**

Outsourced workers presented worse work ability and less favorable sociodemographic and lifestyle aspects among the workers in the study, requiring the maintenance and improvement of work ability in this population.

## Abstract (pt)

**Introdução:**

O trabalho, quando realizado em condições desfavoráveis, pode levar ao desenvolvimento de distúrbios musculoesqueléticos e à diminuição da capacidade para o trabalho.

**Objetivos:**

Identificar diferenças entre três grupos de trabalhadores (professores, técnicos e terceirizados) em relação a perfil sociodemográfico, capacidade para o trabalho, presença de distúrbios musculoesqueléticos e restrição para atividades e correlacionar a presença de distúrbios musculoesqueléticos e a restrição para atividades com cada domínio do índice de Capacidade para o Trabalho.

**Métodos:**

A amostra foi composta por 67 trabalhadores de uma universidade, os quais preencheram um questionário de dados sociodemográficos, o Questionário Nórdico de Sintomas Osteomusculares e o índice de Capacidade para o Trabalho. Foram utilizados estatística descritiva e o teste tau de Kendall para identificar possíveis correlações.

**Resultados:**

Os professores apresentaram aspectos sociodemográficos e de estilo de vida mais favoráveis e maior capacidade para o trabalho, enquanto os terceirizados apresentaram aspectos sociodemográficos e de estilo de vida menos favoráveis, além de menor capacidade para o trabalho. Foi encontrada correlação entre restrição para atividades em apenas um dos domínios do Índice de Capacidade para o Trabalho entre professores. Entre os terceirizados, foram encontradas correlações entre a presença de distúrbios musculoesqueléticos e a restrição para atividades em seis domínios do índice de Capacidade para o Trabalho. Os técnicos administrativos não apresentaram correlações significativas.

**Conclusões:**

Os trabalhadores terceirizados apresentaram pior capacidade para trabalho e aspectos sociodemográficos e de estilo de vida menos favoráveis entre os trabalhadores do estudo, sendo necessárias a manutenção e a melhora da capacidade para o trabalho desta população.

## INTRODUCTION

Musculoskeletal disorders (MSDs) are common conditions in various groups of workers and can have consequences for individuals, but also for households, businesses, and the government. Occupational exposure to different risks can lead to these disorders developing. MSDs are the most common cause of sick leave or disability in Canada, the United States, and the European Union.^1,2^

MSDs are health conditions of the locomotor system, i.e. muscles, tendons, bones, cartilage, ligaments, and nerves. Lesions range from mild and transient symptoms to irreversible and disabling injuries and can be divided into two large groups: cumulative injuries (upper limbs and lower limbs) and spinal injuries.^2^

When performed in unfavorable conditions, work can be detrimental to health, whether due to the organization of work, fast pace, increased working hours, and a shortage of workers, or due to physical demands, with an excessive load on the segments, repetitive movements, and inappropriate postures.^3^ In Brazil, since the 1990s, there has been an increase in the incidence of Work-Related Musculoskeletal Disorders (WMSDs).^4^

Work ability (WA) is a subjective concept that is associated with factors such as physical, mental, and social occupational demands, organizational culture, community, management, and workplace conditions. In Finland, during the 1980s, after noticing the growing aging of the working population, the Finnish Institute of Occupational Health (FIOH) began to develop studies on the main contributing factors to WA. A few years later, following reports from the World Health Organization (WHO) on the subject, the first studies were conducted in Brazil.^5^

Currently, Brazilian studies on WA are centered on nursing and public health, predominantly with health care personnel. Many different factors lead to alterations in WA, such as individual and health-related issues, as well as those related to environmental and organizational working conditions.^6^

In light of this, further research on the subject is needed to encourage debate and raise awareness among managers about the importance of strategies to prevent these issues and promote occupational health, including university workers, as well as encouraging self-care among this population, with a view to improving physical and mental health conditions. Therefore, this study aimed to identify the differences between three groups of workers (professors, administrative technicians, and outsourced workers) in relation to their sociodemographic profile, WA, and presence of MSDs and activity restrictions due to MSDs in the past 12 months in 3 body parts, as well as correlating the presence of MSDs and activity restrictions due to MSDs in the past 12 months with the domains of the Work Ability Index (WAI).

## METHODS

This sample consisted of 67 workers from a public university campus located in southern Brazil, including 27 administrative technicians, 22 professors, and 18 outsourced workers. To define the sample size, a sample calculation was used, estimating a 50% prevalence of musculoskeletal symptoms and considering a 10% sample loss, with a 95% confidence interval and a mean maximum estimation error of 10%.^7^

Workers of both sexes who were literate and able to understand the assessment tools were included in the study, while workers who had worked on campus for less than 3 months, who were not literate, and unable to understand the assessment tools were excluded. Three questionnaires were used to collect the data, the first of which was a sociodemographic questionnaire with functional information related to individual, social, and lifestyle aspects.

All participants were informed about data collection aims and procedures. The study was approved by the institutional research ethics committee with opinion No. 87432118.4.0000.5347. All participants willing to participate in the study signed an informed consent form (ICF), respecting the precepts of research with human beings in Resolution 466/12 of the Conselho Nacional de Saúde (CNS - Brazilian National Council of Health).

The presence of MSDs and activity restrictions due to MSDs in the last 12 months was identified using the Nordic Questionnaire of Osteomuscular Symptoms (QNSO).^8^ This is a self-reporting questionnaire consisting of multiple choice questions regarding the occurrence of disorders in 9 body parts (neck/cervical, shoulders, thoracic spine, lumbar spine, elbows, wrists/hands/fingers, hip/thigh, knee, ankle/foot) and a description of the occurrence of these disorders (pain, discomfort, or numbness).

For this study, 2 of the 4 indices in the questionnaire were used: index 1, for reports of disorders (pain and paresthesia/tingling) in the last 12 months, and index 2, for recording activity restrictions to work, domestic, and leisure activities. Furthermore, in order to analyze the results, the 9 body parts were grouped into 3 in this study: upper limbs (shoulders, elbows, and wrists/hands/fingers), spine (neck/cervical spine, thoracic spine, and lumbar spine), and lower limbs (hip/thigh, knee, and ankle/foot).

The ICT^9^ was used to assess WA. This is an instrument that allows you to assess WA from the worker's self-perception, through 10 questions summarized in 7 dimensions. The sum of the answers indicates the WAI, which can vary between 7 to 49 points. In this range, the score can be classified into 4 different categories: 7 to 27 points: low ability; 28 to 36, moderate ability; 37 to 43, good ability; 44 to 49, excellent ability. In this study, all 4 categories were reduced to 2: low/moderate and good/optimal.

SPSS 20.0 was used for the statistical analysis. Descriptive statistics were used, based on the mean, standard deviation, and frequencies. Kendal's tau test was used to analyze the correlation between WA and MSD and WA and activity restrictions due to MSDs. Only moderate to very strong correlations (0.5 to 0.9) were considered for analysis purposes.

## RESULTS

Sociodemographic data (Table 1) showed that professors had higher education and had a higher family income, in addition to being more physically active than the other groups of workers. MSDs most commonly affected the spine in this group. Similarly, the spine and lower limbs were the parts with the greatest activity restrictions due to MSDs (Table 2). Moreover, professors had the highest WA (Table 3) among the 3 groups. A significant correlation was found among professors between activity restriction due to MSDs in the upper limbs and the current number of diagnosed diseases domain of the WAI (0.564; p = 0.017) (Table 4).

**Table 1:** Frequency of sociodemographic data, musculoskeletal disorders, activity restrictions due to musculoskeletal disorders and work ability

Variable	Professors	Technicians	Outsourced workers
	(±σ)	(±σ)	(±σ)
Age (years)	457 ± 96	49.6 ± 10	39.8 ±10
Weight (kg)	78.4 ±16.2	73.8 ± 16	79.9 ± 18.6
Height (cm)	171.2 ± 8	169.6 ± 7.2	166.9 ±11.2
Time in office (years)	9.9 ± 12	12.9 ±12.5	10.1 ±10.5

	n (%)	n (%)	n (%)
Sex			
Female	11 (61.1)	15 (55.6)	12 (54.5)
Male	7 (38.9)	12 (44.4)	10 (45.5)
Physical activity			
Yes	11 (61.1)	13 (48.1)	8 (36.4)
No	7 (38.9)	14 (51.9)	14(63.6)
Smoking			
Yes	1 (5.6)	5 (18.5)	8 (27.3)
No	17 (94.4)	22 (81.5)	16 (72.2)
MSD (last 12 months)			
Upper limbs	8 (55.6)	15 (55.6)	12 (54.5)
Spine	15(83.3)	21 (77.8)	15(68.2)
Lower limbs	6 (33.3)	2 (7.4)	9 (40.9)
Activity restrictions due to MSD (last 12 months)			
Upper limbs	2 (11.1)	5 (18.5)	9 (40.9)
Spine	11 (61.1)	12 (44.4)	7 (31.8)
Lower limbs	6 (33.3)	6 (22.2)	5 (22.7)
Work ability (points)			
Low or moderate (7-36)	1 (5.6)	4(14.8)	5 (22.8)
Good or excellent (37-49)	17 (94.4)	23 (85.2)	17 (77.2)
Ethnicity/skin color			
White	18 (100.0)	23 (85.2)	17 (77.3)
Black	0 (0.0)	0 (0.0)	3 (13.6)
Brown	0 (0.0)	3 (11.1)	2 (9.1)
Yellow	0 (0.0)	0 (0.0)	0 (0.0)
Indigenous	0 (0.0)	1 (3.7)	0 (0.0)
Age started working (years)			
Under 18	7 (38.9)	11 (40.7)	15 (68.2)
Between 18 and 25	9 (50.0)	15(55.6)	7(31.8)
More than 25	2 (11.1)	1 (3.7)	0 (0.0)
Working time (years)			
Less than 5	0 (0.0)	0 (0.0)	1 (4.5)
Between 5 and 15	3 (16.7)	7 (26.0)	7 (31.8)
Between 15 and 30	6 (33.3)	10 (370)	11 (50.0)
More than 30	9 (50.0)	10 (370)	3(13.6)
Educational background			
Elementary school	0 (0.0)	1 (3.7)	8 (27.3)
High school	0 (0.0)	2 (7.4)	14 (63.6)
Higher education	18 (10.0)	24 (88.9)	2 (9.1)
Monthly family income (MW)			
Between 1 and 2	0 (0.0)	0 (0.0)	15 (68.2)
Between 2 and 10	5 (27.8)	25 (92.6)	7 (31.8)
Above 10	13 (72.2)	2 (7.4)	0 (0.0)

±σ = mean and standard deviation; MSD = musculoskeletal disorders; MW = minimum wage.

**Table 2:** Frequency of musculoskeletal disorders and activity restrictions due to musculoskeletal disorders per body part

	Professors (%)	Technicians (%)	Outsourced workers (%)
MSDs in the spine	83.3	77.8	68.2
Activity restrictions due to MSD in spine	61.1	44.4	31.8
MSDs in upper limbs	44.4	55.6	54.5
Activity restrictions due to MSD in upper limbs	11.1	18.5	40.9
MSD in lower limbs	33.3	7.4	40.9
Activity restrictions due to MSDs in lower limbs	33.3	22.2	22.7

MSDs = musculoskeletal disorders.

**Table 3:** Correlations between the domains of the Work Ability Index and activity restrictions due to musculoskeletal disorders in the last 12 months in professors

WAI domains	Upper limbs		Spine		Lower limbs	
	±σ	ρ	±σ	ρ	±σ	ρ
Number of diagnosed disorders	1.5 ± 0.7	0.564 (p = 0.017)	0.4 ± 0.7	0.104 (p = 0.661)	0.7 ± 0.8	0.282 (p = 0.234)

Bold values indicate significant correlation.

±σ = mean and standard deviation; WAI = Work Ability Index; **ρ** = probability.

**Table 4:** Correlations between the domains of the Work Ability Index and activity restrictions due to musculoskeletal disorders in the last 12 months in outsourced workers

WAI domains	Upper limbs		Spine		Lower limbs	
	±σ	ρ	±σ	ρ	±σ	ρ
Current ability to work	7.4 ± 1.8	-0.399 (p = 0.044)	6.9 ± 1.6	-0.595 (p = 0.003)	7.8 ± 1.6	-0.193 (p = 0.329)
WA prognosis within 2 years	4.7 ± 2.0	-0.718 (p = 0.001)	4.9 ± 1.5	-0.612 (p = 0.004)	6.4 ± 1.3	0.097 (p = 0.650)
Ability to enjoy life	7.3 ± 2.4	-0.441 (p = 0.020)	6.7 ± 1.7	-0.520 (p = 0.006)	9 ± 2.9	0.030 (p = 0.874)
Overall WAI	3 7.1 ± 5.7	-0.508 (p = 0.006)	35.6 ± 4.1	-0.609 (p = 0.001)	40.8 ± 6.6	-0.081 (p = 0.664)

Bold values indicate significant correlation.

**±σ** = mean and standard deviation; WA = Work Ability; WAI = Work Ability Index; **ρ** = probability.

On the other hand, the group of outsourced workers consisted of younger participants with lower education and lower family income (Table 1). In addition to having a higher percentage of sedentary people, smokers, and higher body mass index (BMI) (Table 1), this group was also found as having a higher percentage of MSDs in the lower limbs and activity restrictions due to MSDs in the upper limbs (Table 2), and lower WA (Table 3) when compared to the other groups. There were significant correlations between activity restrictions due to MSD in the upper limbs and prognosis within 2 years in terms of WA and overall WA score (Table 4). Similarly, activity restrictions due to MSDs in the spine showed significant correlations with current WA, the domain for prognosis within 2 years regarding WA, ability to enjoy life, and overall WA score (Table 4). Significant correlations were also found between MSDs in the upper limbs and sick leave, and between MSDs in the spine and the number of diagnosed disorders (Table 5).

**Table 5:** Correlations between the domains of the Work Ability Index and musculoskeletal disorders in the last 12 months in outsourced workers

WAI domains	Upper limbs		Spine		Lower limbs	
	±σ	ρ	±σ	ρ	±σ	ρ
Number of diagnosed disorders	0.8 ± 0.8	0.409 (p = 0.051)	0.8 ± 0.8	0.529 (p = 0.012)	0.1 ± 0.3	0.431 (p = 0.040)
Sick leave	5.2 ± 0.6	-0.635 (p = 0.003)	5.3 ± 0.6	-0.421 (p = 0.049)	5.7 ± 0.4	-0.316 (p = 0.140)

Bold values indicate significant correlation.

**±σ** = mean and standard deviation; WAI = Work Ability Index; **ρ** = probability.

The group of administrative technicians consisted mostly of non-smokers with a lower BMI (Table 1). Among the administrative technicians, there was also a greater presence of MSDs in the upper limbs and lower activity restrictions due to MSDs in the lower limbs (Table 2). However, these findings showed no significant correlation with the presence of MSDs and activity restrictions due to MSDs in the last 12 months in any of the body parts analyzed.

## DISCUSSION

The International Labor Organization (ILO) estimates that 32% of people in Europe, 30% in North America, 21% in Asia, and 17% in Latin America will be older than 55 by 2025^10^. The decline in muscle strength and mass begins around the forties,^11^ which is precisely the mean age of professors in this study who were the group of workers with higher MSDs and activity restrictions due to MSDs in the spine. This can be explained as a result of the ageing process, which can be slowed through exercise, although it is a natural and inevitable process.^11^

Teaching involves multiple activities such as teaching, conducting research, continuous education, and administration. These activities, when performed in unfavorable conditions, place great physical, cognitive, and emotional demands on professors, which can result in physical and mental illnesses and disorders.^12,13^ This can be seen in the significant correlation found between the "number of current disorders" domain of the WAI and activity restrictions due to MSDs in the upper limbs among the professors in this study.

Despite professors had the highest mean age of the three groups, they had the highest WA, which is contrary to other recent studies,^13,15^ which showed an association between older age and lower WA. Nevertheless, WA is also determined by factors other than age, such as sociodemographic and lifestyle factors, which were more favorable in the group of professors in this study.

The so-called social determinants of health in Brazil are the social conditions in which people live and work, such as social, economic, cultural, ethnic/ racial, psychological, and behavioral factors that influence the occurrence of health conditions and risk factors in the population.^16,17^ In addition, studies have shown that there are inequalities in health depending on the social gradient.^18^

This social gradient was less favorable among the group outsourced workers in this study, whose socioeconomic and lifestyle conditions were worse than those of the other groups studied. In the literature, consistent evidence of the association between low WA and lifestyle risk factors, such as high BMI, obesity, and smoking^19,20,21^

Outsourced workers also had more MSDs in the lower limbs and activity restrictions due to MSDs in the upper limbs. This can be explained through the characteristics of outsourced work, which is predominantly manual and uses low-tech tools. In addition, ergonomically inappropriate positions, heavy physical demands, and a high pace of work are some of the main risks to the physical health of these workers.^22,23^

In the present study, outsourced workers had worse physical activity among the 3 groups of workers, and recent findings show that worse WA is associated with low physical activity,^24,25^ while consistent evidence shows that regular physical activity helps to prevent illnesses and improve physical and mental health, also bringing benefits to companies.^25^

Outsourcing and the precarious nature of work has various disadvantages, in addition to the physical ones, due to flexible employment relationships, long working hours with no breaks for rest, and no guarantee of basic rights. Moreover, workers are still subjected to precarious working conditions and have to hold in their psychological suffering to avoid reprisals.^26,27^

Several authors have shown that physical activity programs such as occupational exercises are effective in reducing pain, stress, and helping to correct posture, improving performance and promoting relaxation and well-being, and promoting social relationships at work, which seem to minimize the physical and mental damage caused by work organization.^28,29^

## CONCLUSIONS

In view of the results, it can be concluded that among the 3 study groups, outsourced workers presented worse work ability, sociodemographic, and lifestyle aspects. In addition, significant correlations were found between the presence of MSDs in the last 12 months and the WAI categories, especially among outsourced workers, and between the presence of restrictions on activities due to MSDs in the last 12 months and the WAI categories among outsourced workers and professors.

Therefore, prevention and health promotion strategies are needed to improve the physical and mental health of workers, reorganize their work, and improve their working conditions, so as to maintain and improve their WA.
